# Adaptation of dose-prescription for vestibular schwannoma radiosurgery taking body contouring method and heterogeneous material into account

**DOI:** 10.2340/1651-226X.2025.41924

**Published:** 2025-02-26

**Authors:** Marcus Fager, Michael Gubanski, Åsa Carlsson Tedgren, Hamza Benmakhlouf

**Affiliations:** a Department of Oncology-Pathology, Karolinska Institutet, Solna, Sweden; b Department of Nuclear Medicine and Medical Physics, Karolinska University Hospital, Solna, Sweden; c Department of Radiotherapy, Karolinska University Hospital, Solna, Sweden; d Department of Neurosurgery, Karolinska University Hospital, Solna, Sweden; e Department of Health, Medicine and Caring Sciences, Linköping University, Linköping, Sweden; f Center for Medical Image Science and Visualization, CMIV, Linköping University, Linköping, Sweden

**Keywords:** Adaptation of dose-prescription, convolution algorithm, Gamma Knife radiosurgery, vestibular schwannoma

## Abstract

**Background:**

Majority of vestibular schwannoma (VS) patients have undergone gamma-knife radiosurgery (GKRS) with favorable results. Clinical evidence is derived from doses calculated with a type-a algorithm, which in this case assumes all material to be water. A type-b algorithm (Convolution algorithm [CA]) taking tissue heterogeneity into account is available. Historically, body contour is defined using a 16-point approximation, whereas modern softwares generate the body from Magnetic Resonance Imaging (MRI). The accuracy in dose-calculation algorithms (DCA) and contouring method (CM) will have a significant influence in the relation between clinical outcome and dosimetric data. The objective was to investigate the impact of DCA and CMs on dose distribution while preserving treatment conditions.

**Methods:**

Treatment plans for 16 VS patients were recalculated in terms of DCA and CM. The difference in the dose covering 99% of the VS (D_VS99%_) depending on CM and DCA was estimated. The difference in D_VS99%_ was used to adopt the prescription of new CA-based plans. CA-plans were recalculated to TMR10 to evaluate clinical treatability, as clinical evidence is derived from TMR10-doses.

**Results:**

Both CM and DCA had a significant impact on the dose to VS and surrounding structures. CM altered the doses homogenously by 2.1–3.3%, whereas DCA heterogeneously by 5.0–10.7%. An increase of 9.1[8.1, 10.0]% was found for D_VS99%_ and the CA-plans recalculated into TMR10 resulted in clinically treatable plans.

**Interpretation:**

We conclude that transferring to more modern algorithms that take tissue heterogeneity into account heterogeneously alter dose distributions. This work establishes a safe pathway to adopt prescription dose for VS while preserving clinical treatability.

## Introduction

Gamma-Knife radiosurgery (GKRS) is a neurosurgical technique using multiple, narrow, and non-co-planar Co^60^-beams to treat intracranial targets as malignant diseases (metastases, selected glial tumors), benign tumors (vestibular schwannoma [VS], meningioma, pituitary adenoma), vascular and functional conditions (Arteriovenous Malformation; AVM and trigeminal neuralgia) [1].

High level of accuracy in calculating patient doses, using tissue composition characterized by electron density (ED) acquired by computerized tomography (CT) imaging, has long been essential to external beam treatment planning. In GKRS treatment planning, however, patient doses are calculated by a type-a category algorithm, Tissue-Maximum Ratio (TMR10). TMR10 assumes that all matter inside the surface of the skull, that is the body contour, including air cavities and bones, are all water equivalent. In 2011, Elekta presented their type-b category algorithm, Convolution algorithm (CA), which takes ED into account in calculating the dose, and in 2012 a software update that included the CA was released to the market [2]. The CA has recently been positively evaluated against full Monte Carlo simulations [3, 4]. Also, CA is of the algorithm category recommended to be used in stereotactic treatments by International Commission on Radiation Units and Measurements (ICRU), while TMR10 is not [5, 6].

An algorithm for body contour definition based on MR images was included in the GKRS treatment planning software, Leksell Gamma Plan (LGP), version 11.0. Prior to this, the body contour was generated by 16 measurement points around the patient skull using a specific so-called Skull Scaling Instrument (SSI) or acquired from CT scans, which are not always available since most Gamma Knife centers are MRI-only.

Despite opening up for more accurate patient dose calculations and enabling a fair comparison with other stereotactic techniques such as linear accelerators, the new dose calculation algorithm (CA) is to our knowledge not yet implemented clinically, at least not to a significant extent. One reason might be the absence of established relation between doses calculated by the old (TMR10) and the new algorithm (CA), which is required in order to guarantee a safe transition to the CA, and a recalibration of the prescription doses and tolerance doses for organs at risk (OARs). This is particularly important as the normalized dose-response gradient for tumors and OARs is steep [7] and therefore, the risk of under- or over-dosing may be significant. Furthermore, clinical experience from GKRS treatments obtained over decades’ rests on coupling treatment outcome to doses calculated with TMR10. In transferring from using one dose calculation algorithm to another, it is important to compare dose differences under the same irradiation conditions, which is a well-known practice for other modalities [8–14]. The reason why such comparison has not yet been done for the Gamma Knife is two-fold:

**Table:** 

**Firstly**	Most centers rely solely on MRI for their GKRS treatment planning
**Secondly**	LGP automatically renormalizes the dose distribution by relating the maxdose to the prescription. For example, a prescription of 12Gy to 50% isodose corresponds to maxdose of 24Gy. Therefore, a change from TMR10 to CA will non-uniformly deform the dose distribution including the location of the maximum dose. A simple beam-on time (BOT) comparison is thus not valid, since the original plan field-specific parameters are not preserved.

The two dose calculation algorithms of LGP have been compared by means of differences in BOT [15] (LGP v10.1) and shown to differ significantly, on average with 7.4%, where 1.5% is due to changing the body contour definition from SSI to CT images. Another study [16] made a dosimetric comparison using matched clinical BOTs by iteratively changing the prescription dose and studied the difference between the two algorithms for varying tumor locations.

An issue with BOT analysis is that it only evaluates differences in the treatment time, and therefore at best alludes to a change in D_mean_ and says very little about a change in clinically relevant parameters such as coverage dose, D_max_, V5 Gy, or V10 Gy. Also, a recent study [17] investigated the impact of changing the dose calculation algorithm on a population of 56 patients diagnosed with VS and treated with GKRS. The study focuses on metrics such as D_max_, D_min_, and D_50%_ within the prescribed isodose, and provides a probability map of dose differences between the two algorithms.

Analyzing the changes to the dose within the prescribed isodose is a substitute for analyzing changes to the Clinical Target Volume (CTV) but is dependent on how the dose plan was created and does not evaluate critical clinical parameters connected to clinical outcome, such as coverage, D_mean_ and D_max%_ for the CTV and OAR. Furthermore, the approach by the latter study excludes the step from SSI-generated skull to CT-based skull segmentation. This also highlights the need for retrospective studies in the local clinical environment. Lastly, no studies have tested the results backwards; for example, adapting the prescription dose with the average change in dose coverage and creating plans using CA and analyzing them recalculated to TMR10.

Therefore, the aim of this work is two-fold:

1. Compare the algorithms and body contouring methods for identical irradiation conditions.
2. Generate new CA-plans adopting the change in D_99%_ from (1) to the prescription dose, and then recalculate these new plans with TMR10, while again maintaining identical irradiation conditions, in order to validate their clinical treatability.

## Methods

### Patient selection and imaging description

Patients with VS who underwent GKRS between 2013 and 2018 and had a stereotactic CT and MRI were retrospectively studied. Sixteen patients fulfilling these conditions were identified. These patients consisted of 1 Koos I, 6 Koos II, 5 Koos III, and 4 Koos VI, and they were prescribed to 12 Gy. For the major portion of the patient population (*n* = 11), the patients were treated to an Iso-Dose Line (IDL) of 60%, while the rest of the cases (*n* = 5) were treated to an IDL of 50%. The acquisition parameters for MRI and CT images are described in Table 1. A clinically implemented calibration curve from linac system was used for the CT scans in order to calculate the ED maps. The treatment site for VSs is localized close to the base of skull and the inner ear where dose differences emanating from CT tissue information, or the all-water assumption is expected. Stereotactic T1-MRI were defined by their fiducials and a CT was subsequently co-registered inside LGP to the former. For the registration we applied a ROI over the skull base and excluded the outer ears and nasal cavities from the matching.

**Table 1:** Shows a description of images and their parameters acquired for each patient in the study.

Modality	Manufacturer	Sequence	Slice thickness	Voxel size
MRI	GE 1.5T	FSPGR + Gd	1 mm	1 mm*0.5 mm*0.5 mm
MRI	GE 1.5T	T2 Propeller	3 mm	3 mm*0.5 mm*0.5 mm
CT	GE LightSpeed VCT	120 kV, 230 mA	0.6 mm	0.6 mm*0.5 mm*0.5 mm

CT: computerized tomography.

## Description of workflow

All patients were duplicated and anonymized, with preserved conditions for which the patients were treated and allowed us to manipulate skull volumes. As it is not possible to specify identical irradiation conditions for full treatments within the system, a dedicated software was written in MATLAB 2020a Matworks™ (named ReSamp), by which calculations for partial treatments (one per beam position and dose algorithm type) could be added weighted by the actual BOTs.

The treatment plan, structure set, and the calculated dose with TMR10, were exported as DICOM’s to be used for the external resampling. The dose calculation was always performed with a voxel size of 0.5 mm in each dimension. Since this is how the patient was approved for treatment and the body contour was defined by SSI, it will be referred to TMR10_SSI_ from here on. Once TMR10_SSI_ was exported, a new skull volume was generated based on CT scan and an ED map for CA. Due to the renormalization effect inherently built into LGP, individual dose distributions from each ‘Shot’ during the treatment were needed; therefore, the plan was copied to the number of shots per plan and each copied plan only kept one shot.

All these plans were then exported as DICOM for both TMR10 and CA. The exported individual ‘shot’-dose distribution was then resampled in ReSamp to TMR10_CT_ and CA_CT_ using the equations:


$$TMR10_{CT}=\sum_{i=1}^{n}\;s_{i}\frac{t_{i}^{o}}{t_{i}^{s}}$$

$$CA_{CT}=\sum_{i=1}^{n}\;c_{i}\frac{t_{i}^{o}}{t_{i}^{c}}$$

where *i* corresponds to the shot number, *n* the number of shots, *s_i_* the dose distribution for shot *i* calculated with TMR10, *c_i_* the dose distribution for shot *i* calculated with CA, $t_{i}^{o}$ the time shot *i* had in the plan the patient was treated with, and is the time shot *i* had in the plan for the individual dose distribution calculated with TMR10, $t_{i}^{c}$ and the time shot *i* had in the plan for the individual dose distribution calculated with CA. This made three dose distributions available for comparison:

TMR10_SSI_

TMR10_CT_

CA_CT_

where all plans had matched BOT on each shot. The difference between TMR10_SSI_ and TMR10_CT_ was the impact on the dose distribution caused by the definition of the body contour. The difference between TMR10_CT_ and CA_CT_ was the impact on the dose distribution caused by the different algorithms used to calculate dose.

### Delineation of target and OARs, and Boolean

**Figure 1:**
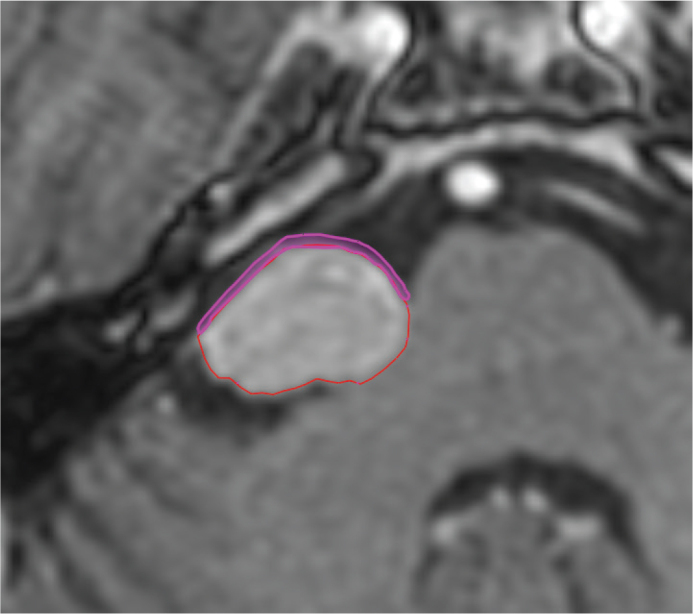
Shows the typical anterior 1 mm expansion we have used in our study as a pseudo-volume in order to estimate the possible differences in doses to the facial nerve.

To achieve consistency in dose to OARs, all these structures were re-delineated as a part of this work by a senior physician in accordance with the latest guidelines [18, 19]. A crucial OAR in VS treatments is the facial nerve (VII) and this is usually difficult to visualize on MR images. However, it is located directly anterior to the target and therefore, a pseudo-volume was created consisting of a 1 mm slab anterior to the target in order to estimate the possible difference in doses to VII; see Figure 1 for an illustration. This pseudo-structure will be referenced to as ‘Anterior’ in the tables and data analysis. Since LGP does not have a function that would allow for a systematic creation of Anterior, the entire re-delineation was performed in an external software Eclipse 15.5. Thereafter the structure set was exported to MICE toolkit (Version 1.1.3, NONPI Medical, Umeå, Sweden) where a Boolean volume was created for each structure in the dimensions of the dose distributions. These Boolean volumes were then used in ReSamp to generate individual DVHs for the structures, for each dose distribution respectively. The generated DHV’s were compared to the exported DVH’s from LGP and Eclipse and the difference was found to be insignificant compared to the differences noticed by changing the algorithms, and since they also were systematic to all cases they were acceptable to use.

### Statistical analysis and evaluation points

Significance was evaluated using paired T-test for the target and Wilcoxon’s signed rank test for OARs where their measurand is not normally distributed. The normality and significance evaluations using standard MATLAB functions were performed in ReSamp for predetermined evaluation points as measurand. These measurands were taken from clinically relevant situations such as dose covering 99% (D_99%_) and coverage (V_12Gy_) of the CTV, and dose constraints for OARs as well as D_mean_ and D_max_ for all structures. A full list of measurands can be found in Table 2.

**Table 2:** Shows the measurand in which evaluations and statistical test were performed.

Structure	Dose [Gy]	Volume [%]
CTV	D_mean_, D_max_ D_99%_	V_12Gy_
Cochlea	D_mean_, D_max_	V_5Gy_
Brainstem	D_max_, D_2%_	
Anterior	D_mean_, D_max_	V_12Gy_

CTV: Clinical Target Volume.

### Replanning with adapted prescription for CA

An adapted prescription dose was found from the change in D_99%_. All patients were retrospectively re-planned using CA (CA_RA_) with the adapted prescription dose. The plans were then recalculated using TMR10 (TMR10_RA_), while preserving the irradiation condition. These plans were then quantitatively evaluated for the metrics described in Table 3, and were also qualitatively evaluated by a senior physician at the clinic to decide if they were clinically treatable, without any modifications.

**Table 3:** Shows our clinical goals. The goals are order in priority order.

Structure	Metric	Measurand
Brainstem	D_0.5cc_ D_0.1cc_ D_0.05cc_	<10 Gy <12 Gy <15 Gy
CTV	V_12Gy_	>99.5%
Cochlea	V_5Gy_	Minimize
Facialis	D_0.1cc_	<12 Gy

CTV: Clinical Target Volume.

## Results

Measurement results, based on the metric described in Table 2, were compared between the three sets of dose distributions: TMR10_SSI_, TMR10_CT_, and CA_CT_. All of the resulting differences can be found in Table 4, where we use a p-value of 0.05 for significance and confidence limits, where applicable, of 95%.

**Table 4:** Reports the results found in this study. TMR10SSI-TMR10CT reports the differences in dose depending on how the body is defined, TMR10CT-CACT reports the differences in dose depending on what algorithm is used while calculating the dose, and TMR10SSI-CACT reports the combined difference taking both body contouring and algorithm used. The table includes 95% confidence intervals and *p*-value for each comparison. D_near-max_ for Anterior was found to be not normally distributed, and are thereby analyzed by Wilcoxon signed rank test, measurand for Dnear-max Anterior are reported as a median.

Structure	Metric	Δ (TMR10_SSI_ – TMR10_CT_)			Δ (TMR10_CT_ – CA_CT_)			Δ (TMR10_SSI_ – CA_CT_)		
		Mean difference		*p*	Mean difference		*p*	Mean difference		*p*
CTV	D_mean_	0.4 [0.3, 0.5] Gy	2.5 [2.1, 2.9] %	<0.001	0.9 [0.8, 0.9] Gy	5.6 [5.2, 6.0] %	<0.001	1.3 [1.2, 1.3] %	8.0 [7.5, 8.5] %	<0.001
	D_max_	0.6 [0.4, 0.7] Gy	2.6 [2.1, 3.2] %	<0.001	1.0 [0.9, 1.1] Gy	5.0 [4.5, 5.4] %	<0.001	1.6 [1.4, 1.7] %	7.5 [7.0, 8.0] %	<0.001
	D_99%_	0.2 [0.1, 0.4] Gy	2.1 [1.1, 3.2] %	<0.030	0.8 [0.7, 0.8] Gy	7.1 [6.4, 7.7] %	<0.001	1.0 [0.9, 1.0] Gy	9.1 [8.1, 10.0] %	<0.001
	V_12Gy_	1.9 [1.4, 2.4] %		<0.001	7.1 [5.5, 8.6] %		<0.001	9.0 [7.1, 10.8] %		<0.001
Cochlea	D_mean_	0.1 [0.1, 0.2] Gy	2.4 [1.4, 3.4] %	<0.001	0.5 [0.4, 0.6] Gy	10.7[10.0, 11.4] %	<0.001	0.6 [0.5, 0.8] Gy	12.9[12.0, 13.8] %	<0.001
	D_max_	0.3 [0.1, 0.5] Gy	3.3 [1.3, 5.3] %	<0.001	0.7 [0.6, 9.2] Gy	9.1 [7.8, 10.4] %	<0.001	1.1 [0.8, 1.3] Gy	12.0[9.7, 14.4] %	<0.001
	V_5Gy_	2.0 [0.1, 4.0] %		<0.040	7.5 [4.2, 10.7] %		<0.001	9.5 [5.1, 13.9] %		<0.001
Brainstem	D_max_	0.1 [0.1, 0.2] Gy	2.4 [1.5, 3.2] %	<0.001	0.4 [0.3, 0.5] Gy	4.8 [4.2, 5.3] %	<0.001	0.6 [0.4, 0.8] Gy	7.1 [6.1, 8.0] %	<0.001
	D_2%_	0.2 [0.1, 0.3] Gy	2.5 [1.8, 3.2] %	<0.001	0.3 [0.2, 0.3] Gy	5.3 [4.9, 5.7] %	<0.001	0.4 [0.2, 0.5] Gy	7.6 [6.8, 8.3] %	<0.001
Anterior	D_mean_	0.3 [0.2, 0.4] Gy	2.3 [1.3, 3.3] %	<0.001	0.9 [0.8, 1.0] Gy	8.1 [7.3, 8.9] %	<0.001	1.2 [1.0, 1.3] Gy	10.2 [9.1, 11.3] %	<0.001
	D_max (median)_	0.5 Gy(WSR)	2.4 % (WSR)	<0.007	1.0 Gy (WSR)	6.5% (WSR)	<0.001	1.5 Gy (WSR)	8.8% (WSR)	<0.001
	V_12Gy_	4.8 [2.8, 6.8] %		<0.001	11.3 [6.9, 15.7] %		<0.001	16.1 [10.0, 22.2] %		<0.001

SSI: Skull Scaling Instrument; CTV: Clinical Target Volume.

### TMR10_SSI_ versus TMR10_CT_

It was found that changing the definition of body contour from SSI to MR-based skull segmentation while keeping all other plan metrics constant, significantly decreased the dose for almost all evaluated parameters with slightly more than 2–3%. The only measurand that differs from this is V12 Gy of Anterior which decreased by 4.8%.

### TMR10_CT_ versus CA_CT_

While keeping the definition of the body contour constant and evaluating the impact of the calculation algorithm solely, it was found that CA significantly lowered the estimated doses for all the evaluated parameters when compared to TMR10. For the CTV the D_mean_, D_max_, D_99%_, and V_12Gy_, decreased with 5.6, 5.0, 7.1, and 7.1%, respectively. For the cochlea it was found that the max, mean, and V_5Gy_, the doses decreased with 9.1, 10.7, and 7.5%, respectively, Anterior the D_mean_, D_max_, and V_12Gy_ decreased with 8.1, 6.5, and 11.3%, respectively, and for Brainstem the D_max_ and D_2%_ decreased with 4.8, and 6.5%, respectively.

### TMR10_SSI_ versus CA_CT_

When combining the effect of changing both the body contour method and calculation algorithm, it was found that CA_CT_ significantly lowered the estimated doses for all the evaluated parameters when compared to TMR10_SSI_. For the CTV, the D_mean_, D_max_, D_99%_, and V_12Gy_ decreased with 8.0, 7.5, 9.1, and 9.0%, respectively. For the cochlea, it was found that D_mean_, D_max_, and V_5Gy_, decreased with 12.9, 12.0, and 9.5%, respectively, Anterior the D_mean_, D_max_, and V_12Gy_ decreased with 10.2, 8.8, and 16.1%, respectively, and Brainstem the D_max_ and D_2%_ decreased with 7.1 and 7.6%, respectively.

### Replanning with adapted prescription for CA

Since the D_99%_ parameter for the CTV decreased by an average of 1.0 Gy, the prescribed dose in CA_RA_ replans was adapted with the same amounts to 50/60% IDL. The TMR10_RA_ plans were all deemed as clinically viable to treat a patient with by the attending physician, with sufficient target coverage and OAR sparing.

Evaluating each patient against the metrics our clinic has for VS, each plan either passes on every metric, or fails for Cochlea. However, the patients who fail for cochlea, the VS is directly adjacent, and for the original plans for these patients the target coverage was prioritized over cochlea sparing. A summary of quantitative evaluation can be found in Table 5 and shows that on the cohort base TMR10_RA_ is within the clinical bounds.

**Table 5:** Reports the TMR10_RA_ doses and 95% confidence intervals, which are planned in Convolution algorithm with the adjusted prescription and tolerances found in Table 3 and then recalculated to TMR10 in order to be able to analyze them with respect to current clinical practice.

Structure	Metric	TMR10_RA_
CTV	D_mean_	17.2 [16.9, 17.5] Gy
	D_max_	22.9 [22.4, 23.5] Gy
	D_99%_	12.4 [12.2, 12.7] Gy
	V_12Gy_	99.7 [99.4, 100] %
Cochlea	D_mean_	3.9 [2.7, 5.1] Gy
	D_max_	7.5 [4.4, 10.6] Gy
	V_5Gy_	21.6 [0.1, 43.3] %
Brainstem	D_max_	6.1 [2.3, 9.8] Gy
	D_2%_	1.4 [0.5, 2.3] Gy
Anterior	D_mean_	11.5 [11.0, 12.1] Gy
	D_max_	16.1[15.0, 17.2] Gy

CTV: Clinical Target Volume.

## Discussion

Since CA calculates dose distributions from the patient anatomy and not homogenous water volumes, a change to the new algorithm would further increase precision in GKRS and improve the accuracy in estimating tumor control and normal tissue complications. It would also open up for a more accurate comparison with other stereotactic techniques and allow for a safer transfer of knowledge between different treatment modalities. There is an increasing interest in extending GKRS to diagnoses such as larger brain tumors that require fractionated treatment schedules and are mostly treated with conventional linear accelerators. For an accurate transfer of knowledge of dose-response relationships, similar studies with larger patient cohorts are warranted for standardized treatments with well-known clinical outcomes. The prescription dose to the target and OAR tolerance doses in single-fraction Gamma Knife radiosurgery is well-established in Gamma Knife radiosurgery. As previously stated, these doses are to a great extent based on doses calculated using TMR10, a type-a dose calculation algorithm. In order to make these doses transferable to other modalities, this work stresses the need to convert the TMR-10 based doses to type-b dose-calculation algorithms (DCA) before a unification of doses between Gamma Knife and other modalities using type-b algorithms. For example, Goldbrunner et al. [20] recommend single-fraction doses to VS between 11 and 14 Gy for different modalities (e.g. linear accelerators, Gamma Knife, or Cyberknife). It is stressed that for a fair clinical comparison between these modalities, or extrapolation of clinical data from Gamma Knife to the other cited modalities, adapting the doses suggested in this work is warranted. We theorize that our findings are transferable to other Gamma Knife sites that have high conformity dose planning for their VS; however, we strongly urge each site to thoroughly test the CA_RA_ -> TMR10_RA_ step before clinical implementation. We theorize that if the dose plans are less conform and VS do not have a steep gradients at its border, the results might be different, and this is a scope for further research.

As shown in this retrospective study, changing from TMR10_SSI_ to CA_CT_ but preserving the dose prescription, that is, 12 Gy to 50/60% IDL, will effectively increase the mean dose to the target by 8%, and cochlea by 12.9% whereas V_12Gy_ will increase with 16.1% to the Anterior structure. To account for all this, the prescription should be adjusted by 9.1%. If a treatment plan is calculated by CA, this could optimally be done by adjusting the prescription dose from 12 to 11 Gy (rounded up from 10.9 Gy). At the same time, the OAR dose limits for Cochlea should preferably be adjusted by replacing V_5Gy_ with V_4.5Gy_. Not adjusting prescription and tolerance doses when changing the calculation algorithm could potentially increase the risk of cochlea and facial nerve toxicity and peritumoral edema, due to the expanded dose distribution.

The largest differences occurred in the measurand surrounding the steepest dose gradients such as D_99%_ dose to the CTV, that decreased with 9.1%, compared to a decrease of the mean dose with 8.0%. This is also seen in the differences of the mean dose or the V_12Gy_ of the Anterior structure that differs by 10.2 and 16.1%. The Anterior structure itself is by its own definition the steep falloff anteriorly of the target.

The change between the two algorithms (TMR10_CT_ and CA_CT_) with respect to D_mean_ to the target was 5.6 [5.2, 6.0] %, and this is comparable with previous studies [15] that found a change in the treatment time of 5.9 [2.1, 8.8] %, and another study [21] 4.2–8.1% depending on the target site. The most recent study [17] defines the target as the ‘50% iso dose line’, which is an approximation in itself. For the latter structure, the article defines a measurand, D_50%_, as ‘percentage of target receiving 50% prescribed dose’. This measurand could be compared to V_12Gy_ in the study, and the results, 7.1 [5.5, 8.6] %, are then slightly lower than their results of 11.3% but fall within their confidence intervals [4.7, 16.1] %.

A recent important and useful study connects the probability of hearing preservation in GKRS treatments to the mean and max BED to Cochlea [22]. The BED is calculated from TMR10-based dose distributions, where the mean/max dose can vary with 10%/9% compared to CA-based dose distributions as shown in this work. We urge caution in using these outcome predictions outside of TMR10 dose distributions, this since BED-calculations are non-linear, and therefore the probability of hearing decline cannot easily be translated to a type-b calculated dose distribution.

In this work we explore whether changes in coverage can be used to adopt the prescription dose for a treatment where CA is used whilst planning the treatment and then analyze that dose distribution using TMR10. We have found that if we use a CA prescription of 11 Gy to IDL of 50/60% and then recalculate it to TMR10, this leads to clinically acceptable plans. This confirms that the method is viable and accurate; however, we recommend that a bridging period, which is already standard in conventional RT, should always be implemented when changing the DCA in LGK in order to discover potential need of re-adjusting the prescription or tolerance doses in order to maintain the treatment efficacy without negatively altering the incidence of toxicity.

These kinds of recalculations are standard to perform in EBRT and are routinely done during an upgrade or change of the dose algorithm. The evaluation is fairly easy to perform and analyze within the TPS for EBRT. This is however not the case for LGP, where side-by-side evaluation or differential evaluation is not available in the TPS, and therefore you must use external software in order to analyze and compare the differences between the dose distributions. Only in later software versions of LGP is it possible to export dose distributions calculated with matched spot positions in CA_CT_ but this requires TMR10_CT_; therefore, it is not trivial to connect clinical data from TMR10_SSI_ to CA_CT_ and thus the method presented in this work is necessary. The method used in this work is, however, time-consuming, as for example a plan with 20 shots takes 2.5 h to export, which makes an analysis of a large patient material very protracted.

Finally, it is important not to extrapolate the result of this work to other treatment sites. This is particularly important considering the fact that VS is located in the skull base. SSI builds the skull by using 16 points and extrapolates the skull in the inferior direction which fails to simulate the decreasing circumference of the most inferior parts of the skull (among this the skull base). This suggests that the dose differences from the SSI and CT-based skull are assumed to be larger in the skull base compared to the areas superior to this. Therefore, one must not generalize the result of this work to all treatment sites. It is stressed that other sites must be independently analyzed, which is the scope for future research.

## Conclusions

We conclude that our results agree with previously reported results, but also provide new dimensions by reporting differences in measurands with clinical relevance such as the CTV, actual differences in the estimated D_mean_, D_99%_, and D_max_ which have not been reported previously. This work particularly warns for the increased risk of overdosing the OARs if changes in calculation algorithms are not accompanied with adaptation in dose prescriptions. Also, we conclude that using the difference in CTV D_99%_ between the two algorithms can be safely used to adjust the prescription of a CA-based plan and will produce a clinically viable plan when recalculated to TMR10, with preserved CTV coverage and sparing of OARs.

## Data Availability

All data generated by this project are included in this article. The software used was designed to output results in the format presented in Table 4. It does not provide absolute values of the measurands, nor does it export a dose distribution for each scenario.

## Previous presentations

M Fager et al. – Dosimetric Comparison between two dose calculation algorithms available in Gamma knife planning system. AAPM 2020, Online, 12 – 16 July, 2020, Poster Presentation.
